# Supplementary material for: Regulation of alternative splicing at the single‐cell level
Source: Mol Syst Biol. 2015 Dec 28;11(12):845. doi: 10.15252/msb.20156278 (PMC4704489; doi:10.15252/msb.20156278)
Supplement: Supplementary file 1 — Appendix [file MSB-11-845-s001.pdf]

Appendix

Regulation of Alternative Splicing at the Single-Cell Level

Lior Faigenbloom[1,†], Nimrod D. Rubinstein[2,4,†], Yoel Kloog[1], Itay Mayrose[3], Tal Pupko[2,*], Reuven Stein[1,*]

[1]The Department of Neurobiology, George S. Wise Faculty of Life Sciences, Tel Aviv University, Israel

[2]The Department of Cell Research and Immunology, George S. Wise Faculty of Life Sciences, Tel Aviv University, Israel.

[3]The Department of Molecular Biology and Ecology of Plants, George S. Wise Faculty of Life Sciences, Tel Aviv University, Israel.

[4]Current address: Department of Molecular and Cellular Biology, Harvard University, Cambridge, MA 02138, USA

[*]To whom correspondence should be addressed. E-mail: talp@tau.ac.il (T.P.), reuvens@post.tau.ac.il (R.S.)

[†]These authors contributed equally to this work.

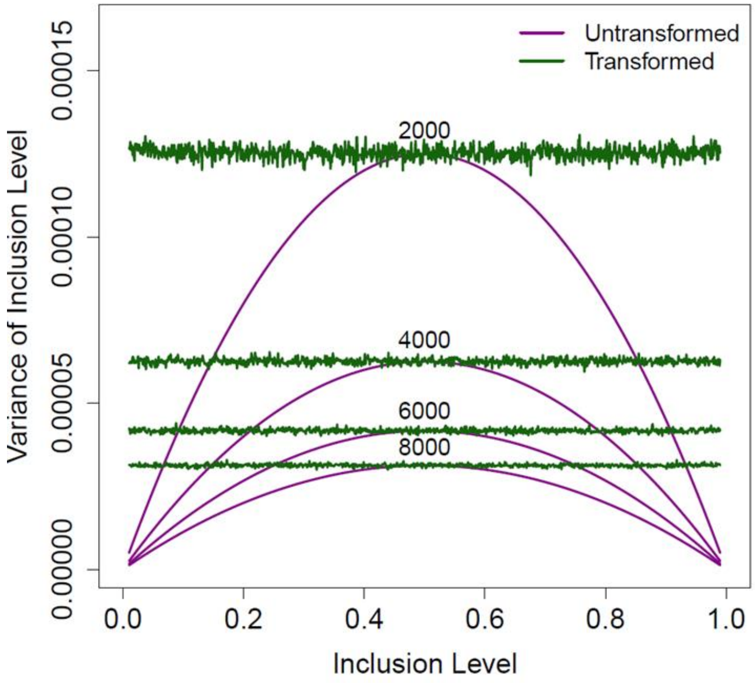

Appendix Figure S1. Variance of inclusion levels as a function of inclusion and expression levels.

For each success probability (i.e., inclusion level) along the (*x*-axis) the number of successes (i.e., included isoforms) was drawn from a binomial distribution for 2,000, 4,000, 6,000, and 8,000 trials (i.e., expression levels of included and skipped isoforms, as indicated in the figure) 10,000 times. The maximum likelihood estimates of the inclusion levels were then computed as the fraction of included isoforms out of expression level. The *y*-axis shows the sample variances across the 10,000 untransformed inclusion-level estimates (magenta curves) and transformed (variance-stabilizing transformation as described above, green points) inclusion-level estimates.

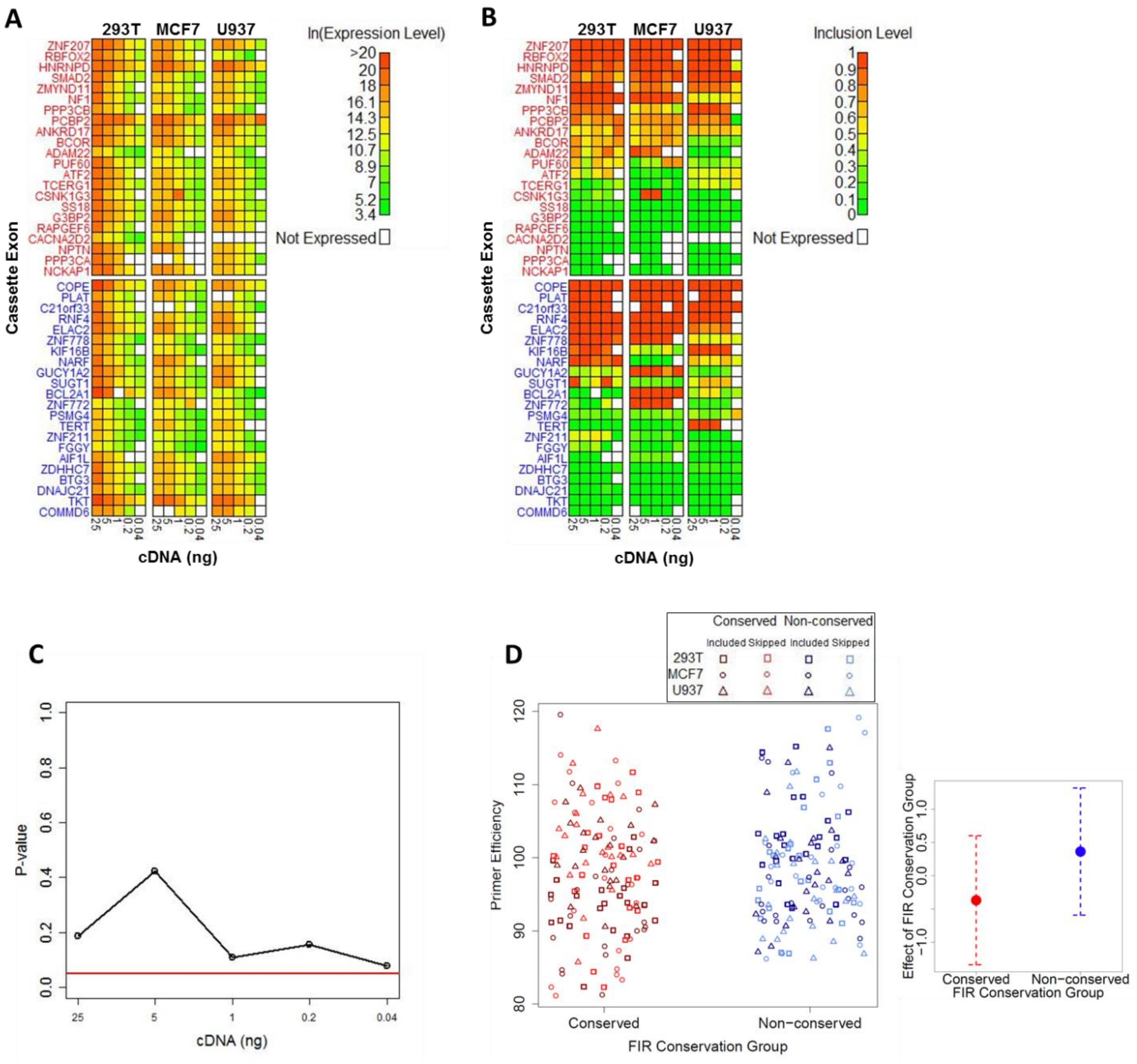

Appendix Figure S2. RT-qPCR data of the bulk RNA.

(**A**) Heat maps of the expression levels (in $\ln(Et)$ units) of included and skipped isoforms of the cassette exons. Different cDNA dilutions were prepared from the pooled RNA isolated from the first round of sub-cloning of the three cell types. Gene names of the conserved (red) and non-conserved (blue) cassette exons are in rows and single-cell samples of each of the three cell types are in columns.

(**B**) Heat maps of the estimated inclusion levels of the cassette exons.

(C) Testing whether the deviation of inclusion levels estimated from the RT-qPCR data of the bulk RNA and mean inclusion levels of the single-cell data are significantly different from zero (*P*-value in the *y*-axis) for each cDNA dilution along the *x*-axis. The horizontal red line indicates the $P = 0.05$ cutoff.

(D) Primer-pair efficiencies are not different between the two FIR conservation groups, accounting for cell type and isoform (included and skipped).

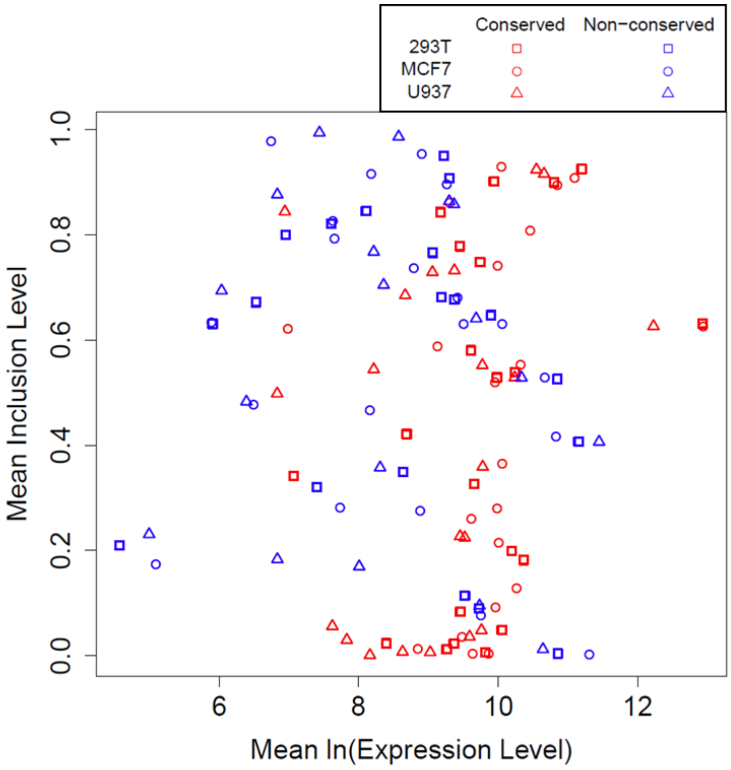

Appendix Figure S3. Mean inclusion levels versus mean expression levels.

Each point represents the mean expression level of the included and skipped transcripts of each of the cassette exons (in $\ln(Et)$ units, x-axis) and the corresponding mean inclusion level (y-axis) across all single-cell samples for each cassette exon in the RT-qPCR data.

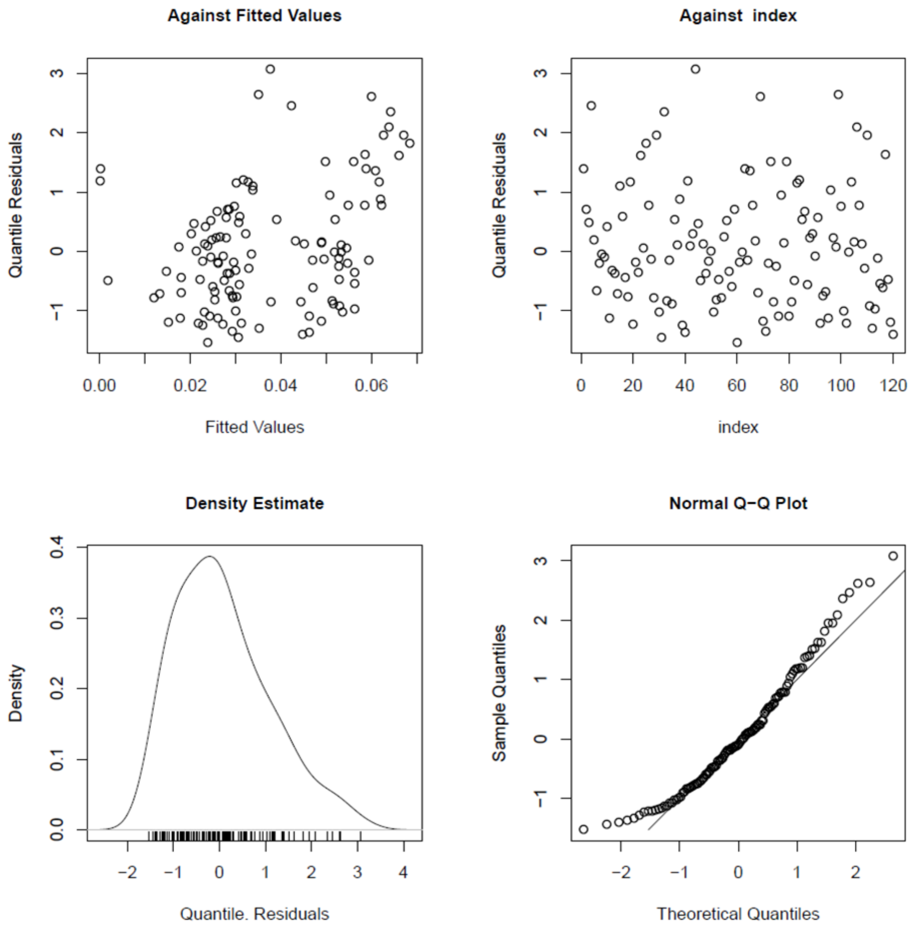

Appendix Figure S4. Diagnostic plot for the GLMM fitted to the single-cell RT-qPCR data.

Top left is the plot of residuals versus the fitted values. Top right is the plot of residuals versus an index. Bottom left is the kernel density estimate of the residuals. Bottom right is the QQ-normal plot of the residuals.

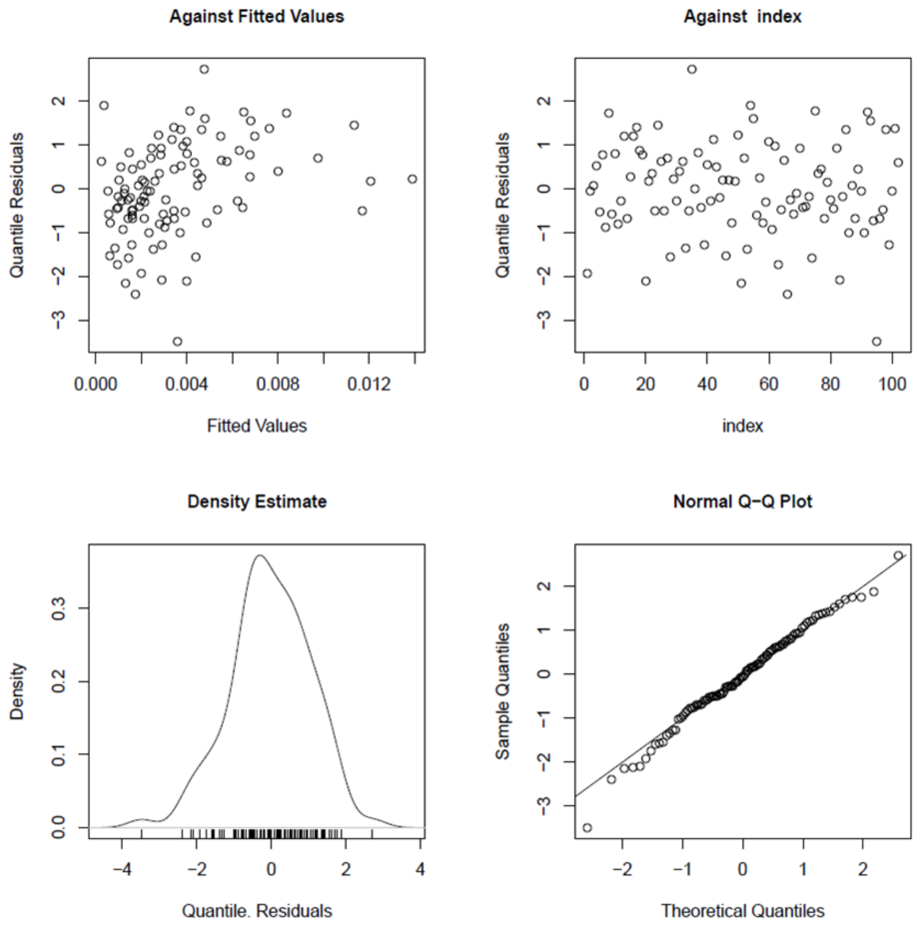

Appendix Figure S5. Diagnostic plot for the GLMM fitted to the control experiment bulk RT-qPCR data.

Top left is the plot of residuals versus the fitted values. Top right is the plot of residuals versus an index. Bottom left is the kernel density estimate of the residuals. Bottom right is the QQ-normal plot of the residuals.

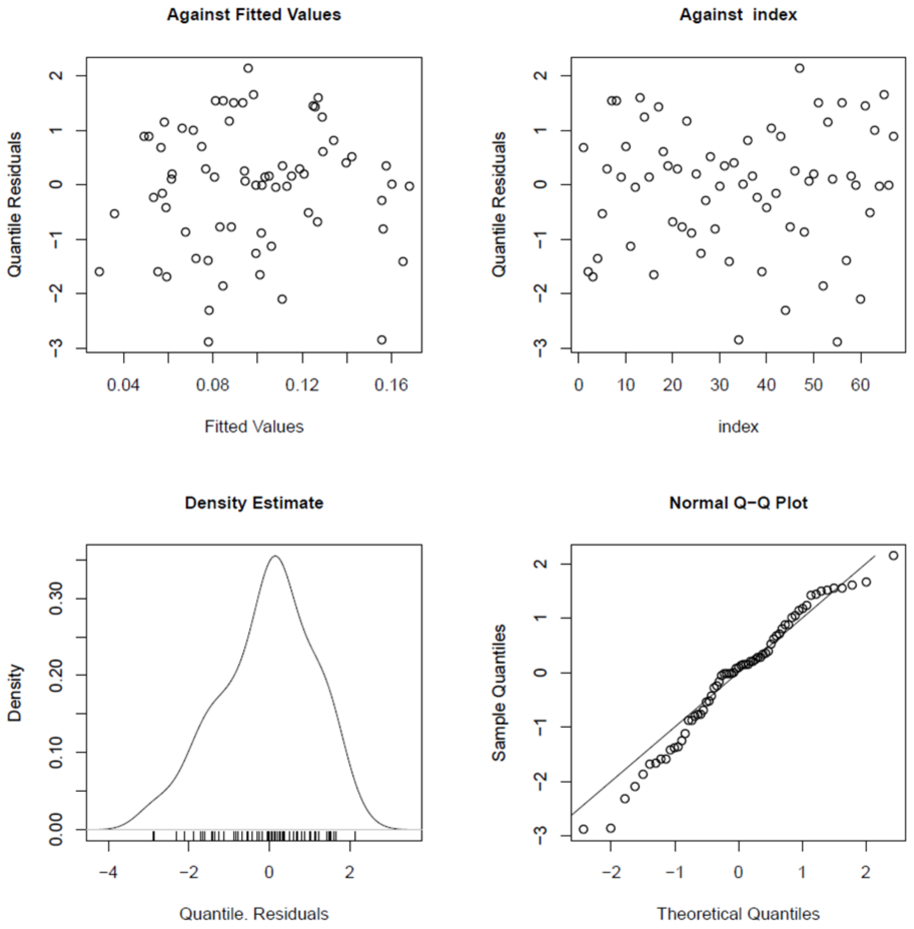

Appendix Figure S6. Diagnostic plot for the GLMM fitted to the hESC data.

Top left is the plot of residuals versus the fitted values. Top right is the plot of residuals versus an index. Bottom left is the kernel density estimate of the residuals. Bottom right is the QQ-normal plot of the residuals.

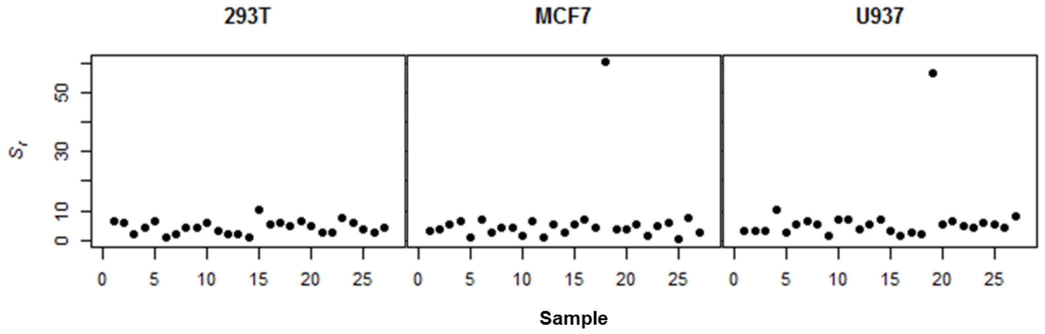

Appendix Figure S7. Detection of outlying failed single-cell samples.

The *y*-axes represent the failure-of-expression scores. The two outlying single-cell samples in cell types MCF7 and U937 were filtered from all subsequent analyses.

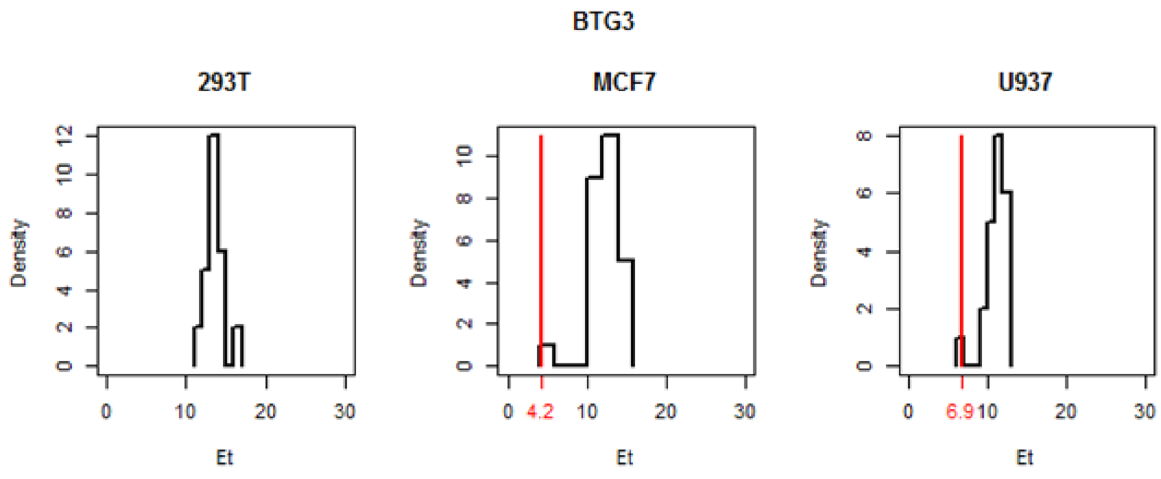

Appendix Figure S8. Filtering of single-cell samples that fall below the limit of detection.

Histograms of the *Et* values of an example skipped isoform (of the alternative exon of the BTG3 gene from the non-conserved group) along all single-cell samples for each of the three cell types. The red lines in the histograms corresponding to MCF7 and U937 represent the limit of detection. Samples below these limits were filtered from all subsequent analyses.

A

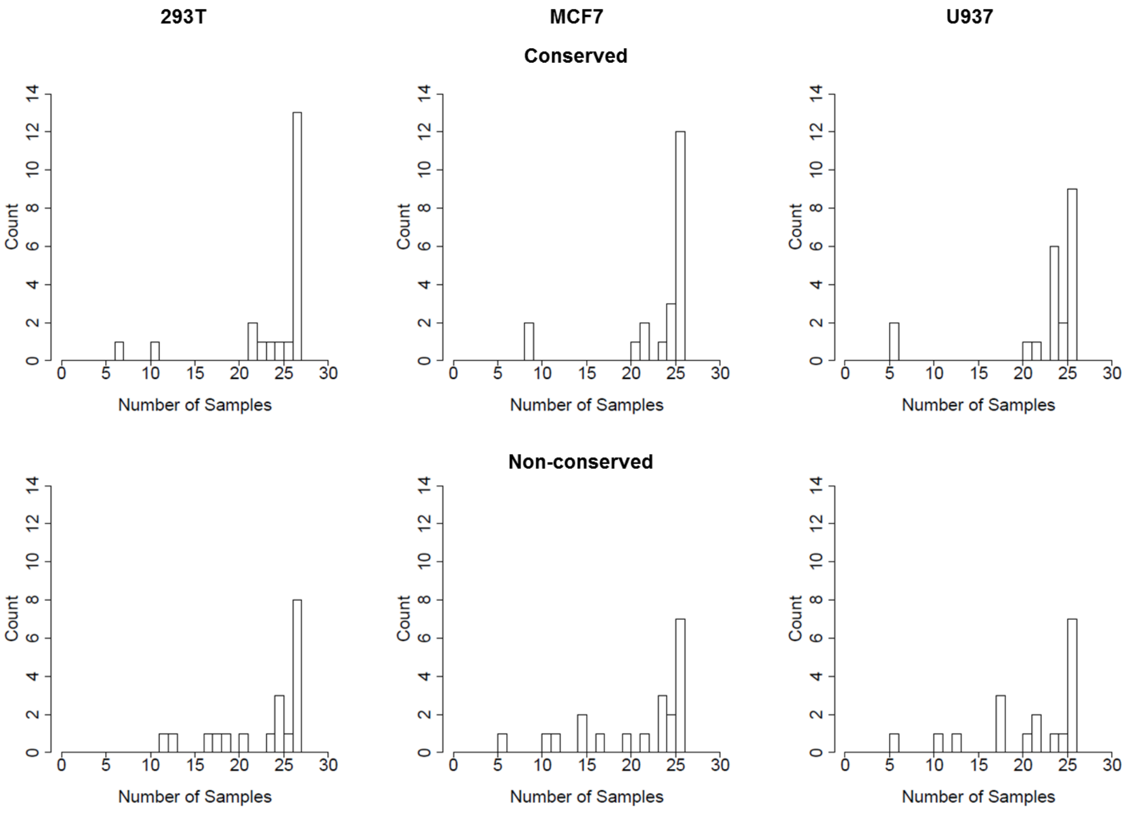
293T
MCF7
U937
Conserved
Non-conserved
Count
Number of Samples

B

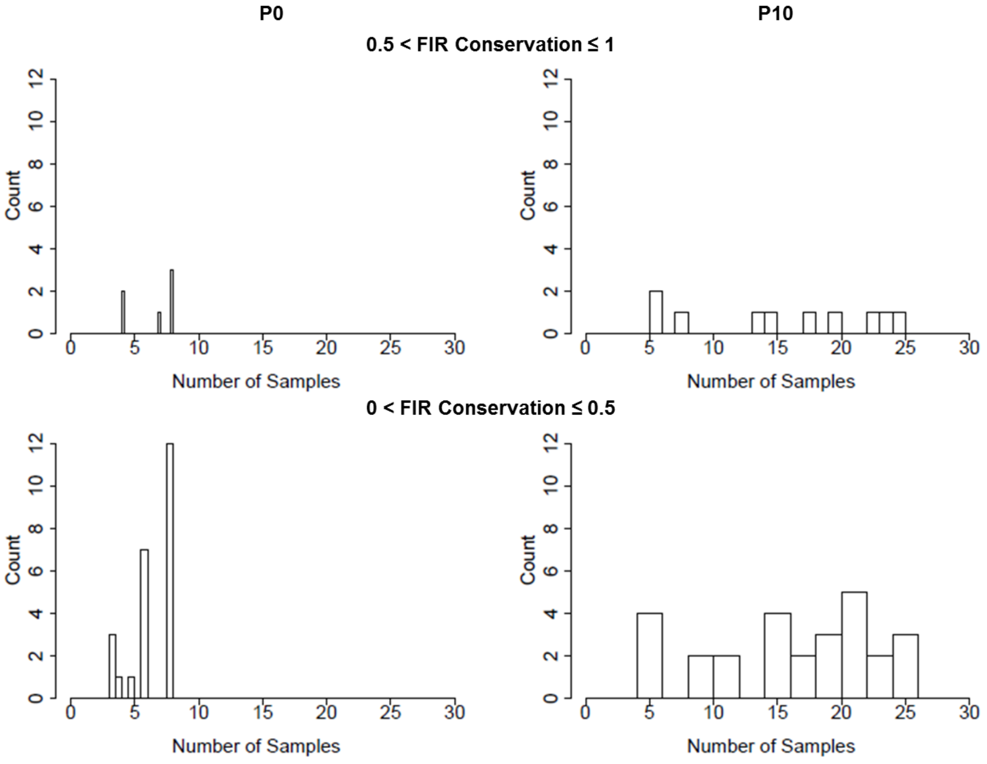
P0
P10
0.5 < FIR Conservation ≤ 1
0 < FIR Conservation ≤ 0.5
Count
Number of Samples

Appendix Figure S9. Histograms of the numbers of the single-cell samples.

(A) Histograms of the numbers of the single-cell samples for all cassette exons in the RT-qPCR experiment (subsequent to all filtering steps), for each of the three cell types in the conserved cassette-exon group (top panel) and non-conserved cassette-exon group (bottom panel).

(B) Histograms of the numbers of the single-cell samples for all differentiation-switch cassette exons in the hESC data (subsequent to all filtering steps), for each of the two cell populations in conserved differentiation-switch cassette exons ($0.5 < \text{FIR conservation score} \leq 1$, top panel) and non-conserved differentiation-switch cassette exons ($0 \leq \text{FIR conservation score} \leq 0.5$, bottom panel).
