# Supplementary material for: Regulation of alternative splicing at the single‐cell level
Source: Mol Syst Biol. 2015 Dec 28;11(12):845. doi: 10.15252/msb.20156278 (PMC4704489; doi:10.15252/msb.20156278)
Supplement: Supplementary file 2 — Expanded View Figures PDF [file MSB-11-845-s002.pdf]

Expanded View Figures

Figure EV1. Control experiment of bulk RNA RT–qPCR data.

A Heat maps of the expression levels (in ln(E_t) units) of included and skipped isoforms of the cassette exons. Gene names of the conserved (red) and non-conserved (blue) cassette exons are in rows, and bulk RNA samples of each of the three cell types are in columns.

B Heat maps of the estimated inclusion levels of the cassette exons.

C Dependence of the variance of the cassette exon inclusion levels (*y*-axis) on their mean inclusion levels (*x*-axis) in the bulk RNA RT–qPCR data. This dependence resembles the expected dependence under the assumption that inclusion or exclusion of a cassette exon is a Bernoulli experiment (Appendix Fig S1).

D High expression levels of the included and skipped isoforms increase the precision of their inclusion levels. The left panel shows the variance of the transformed inclusion levels as a function of the cassette exon expression levels. The right panel shows the effect (solid line) of expression level on the variance of inclusion levels as determined by the GLMM analysis, along with its standard error (dashed lines).

Source data are available online for this figure.

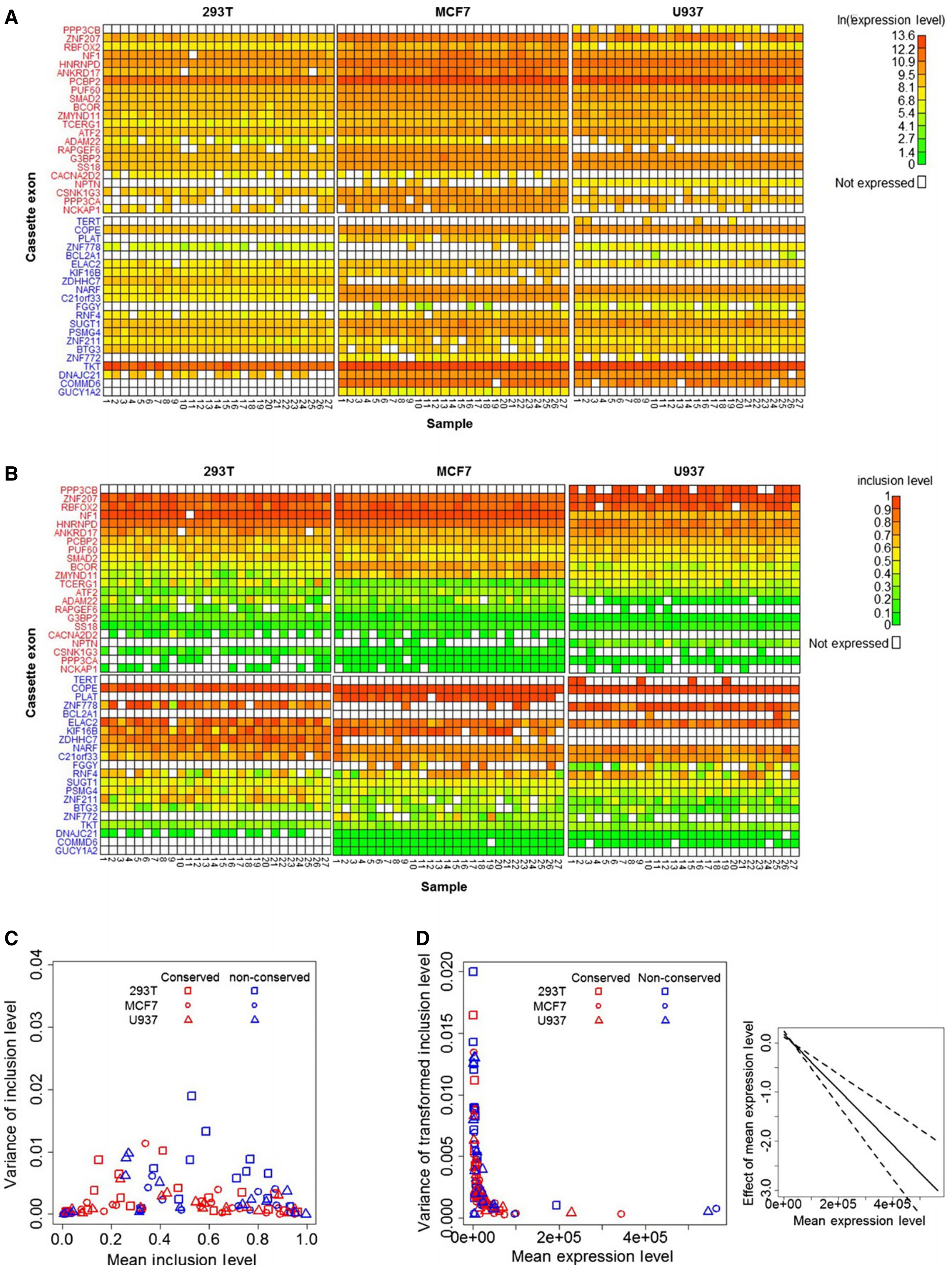

Figure EV1.

© 2015 The Authors
